# RETRACTION: Downregulation of hsa‐miR‐135b‐5p Inhibits Cell Proliferation, Migration, and Invasion in Colon Adenocarcinoma

**DOI:** 10.1155/genr/9859242

**Published:** 2026-09-29

**Authors:** Genetics Research

RETRACTION: Y. Zhang, W. Zhang, W. Xia, J. Xia, and H. Zhang, “Downregulation of hsa‐miR‐135b‐5p Inhibits Cell Proliferation, Migration, and Invasion in Colon Adenocarcinoma,” *Genetics Research* 2022, no. 1 (2022): 2907554, https://doi.org/10.1155/2022/2907554.

The above article, published online on 21 October 2022 in Wiley Online Library (https://wileyonlinelibrary.com), has been retracted by agreement between the Associate Editor, Neetu Rawal and John Wiley & Sons, Ltd. The retraction has been agreed after the authors initially requested a correction to Figure 4 (d). Panel labels below are reproduced exactly as they appear in the original figure, including the original spelling.

Specifically, the panels “hsa‐miR‐135b‐5p inhibitor–migration” and “inhibitor negative control group–invasion,” had been duplicated during figure preparation.

Further investigation by the journal revealed unexpected similarities between the panels “control–migration” and “control–invasion” and the panels “inhibitor negative control–migration,” “inhibitor negative control–invasion” and “hsa‐miR‐135b‐5p inhibitor–migration.”

The authors were unresponsive to the journal’s requests for the original images and underlying raw data. As such, the results cannot be considered as reliable and the article is therefore retracted.

